# Stereotactic Body Radiotherapy for the Treatment of Oligometastases Located in the Peritoneum or in the Abdominal Wall: Preliminary Results from a Mono-Institutional Analysis

**DOI:** 10.3390/jpm15070312

**Published:** 2025-07-14

**Authors:** Francesco Cuccia, Salvatore D’Alessandro, Marina Campione, Vanessa Figlia, Gianluca Mortellaro, Antonio Spera, Giulia Musicò, Antonino Abbate, Salvatore Russo, Carlo Messina, Giuseppe Carruba, Livio Blasi, Giuseppe Ferrera

**Affiliations:** 1 Radiation Oncology, ARNAS Civico Hospital, 90100 Palermo, Italy; vanessa.figlia@arnascivico.it (V.F.); gianluca.mortellaro@arnascivico.it (G.M.); antonio.spera@arnascivico.it (A.S.); giuseppe.ferrera@arnascivico.it (G.F.); 2 Clinical Research, ARNAS Civico Hospital, 90100 Palermo, Italy; marina.campione@arnascivico.it (M.C.); giuseppe.carruba@arnascivico.it (G.C.); 3 Department of Biostatistics, Kore University, 94100 Enna, Italy; 4 Gynecology Oncology, ARNAS Civico Hospital, 90100 Palermo, Italy; giulia.musico@arnascivico.it (G.M.); antonino.abbate@arnascivico.it (A.A.); 5 Medical Oncology, ARNAS Civico Hospital, 90100 Palermo, Italy; salvatore.russo1@arnascivico.it (S.R.); carlo.messina@arnascivico.it (C.M.); livio.blasi@arnascivico.it (L.B.)

**Keywords:** stereotactic radiotherapy, peritoneal nodules, oligometastases, abdominal wall

## Abstract

**Purpose/Objective(s):** Peritoneal carcinosis can occur in several gastrointestinal or gynecological malignancies and its prognosis is usually poor. With the advent of more effective systemic agents, the overall survival of metastatic patients has been revolutionized and isolated peritoneal or abdominal wall metastases might benefit from local treatments; Stereotactic Body Radiotherapy (SBRT) might be considered in selected patients with oligometastatic presentation. **Materials/Methods:** Oligometastases were defined according to recent ESTRO/EORTC consensus. Inclusion criteria were as follows: ECOG PS ≤ 2, written informed consent, up to five lesions to be treated at the same time, patients treated with radiotherapy schedules applying minimum 6 Gy per fraction. The primary endpoint of the study was local control (LC); acute and late toxicity, distant progression-free survival (DPFS), time-to-next systemic treatment (TNST), polymetastatic-free survival (PMFS) and overall survival (OS) were secondary endpoints. Toxicity was assessed according to CTCAE criteria v5.0. Statistical associations between clinical variables and outcomes were assessed using Fisher’s exact test, and Kruskal–Wallis test, as appropriate. Survival outcomes were estimated using the Kaplan–Meier method and compared using the log-rank test. **Results:** Between April 2020 and September 2024 a total of 26 oligometastatic lesions located in the peritoneum or in the abdominal wall detected in 20 patients received SBRT with Helical Tomotherapy. All cases have been assessed by a multidisciplinary team. Only in three patients out of twenty did more than one lesion receive SBRT: two lesions in two patients, and five lesions in a single case of colorectal cancer with ongoing third-line systemic treatment. Median total dose was 30 Gy (27–35 Gy) in five fractions (3–5). The most frequent primary neoplasm was ovarian cancer in 14/20, endometrial in 2/20, while the remaining were colorectal, vaginal, pancreatic and non-small cell lung cancer. Four lesions were located in the abdominal wall, while the remaining twenty-two were located in the peritoneum. Concurrent systemic therapy was administered in 18/20 patients. With a median follow-up of 15 months (range, 6–59), our 1-year LC was 100%, while 1-year DPFS, PMFS, TNTS and OS rates were 54%, 69%, 61% and 83%, respectively. Abdominal wall location and treatment of a subsequent oligometastatic recurrence with a second course of SBRT were both significantly associated with improved OS (*p* = 0.03 and *p* = 0.04, respectively). No G ≥ 3 adverse events occurred. **Conclusion:** Our preliminary data support the use of SBRT in selected cases of oligometastatic disease located in the peritoneum or in the abdominal wall with excellent results in terms of tolerability and promising clinical outcomes.

## 1. Introduction

### 1.1. Peritoneal Carcinomatosis: Challenges and Emerging Treatments

Peritoneal carcinomatosis is a significant cause of mortality for patients with gastrointestinal cancers and frequently affects those diagnosed with advanced gynecological malignancies. This condition can manifest either as a synchronous peritoneal spread (diagnosed at the same time as the primary tumor) or as a metachronous presentation (occurring after initial treatment) [1,2].

For both types of cancers, cytoreductive surgery combined with intraperitoneal chemotherapy has markedly improved disease control rates, yielding promising results. Despite these advancements, the prognosis remains poor, especially for patients with colorectal primary tumors. Furthermore, the occurrence of pelvic side wall recurrences presents a considerable therapeutic challenge for the multidisciplinary team [3,4,5].

### 1.2. Advances in Systemic Therapy

The advent of novel systemic agents, such as immunotherapy, bevacizumab, and PARP-inhibitors, often used in conjunction with conventional chemotherapy, has also significantly improved the prognosis for patients with peritoneal carcinomatosis [6,7,8,9]. These positive outcomes have reshaped our understanding of the metastatic patient, emphasizing the importance of prolonging effective lines of systemic therapy. This approach aims to delay switching to new systemic agents, thereby contributing to an overall survival advantage [10].

Consequently, when disease progression is localized and potentially amenable to local treatments, a thorough multidisciplinary patient selection process can lead to remarkable clinical outcomes [11].

### 1.3. The Role of Stereotactic Body Radiotherapy (SBRT)

In this evolving landscape, Stereotactic Body Radiotherapy (SBRT) has been explored in various contexts of oligometastatic disease. SBRT serves as a valuable therapeutic tool that clinicians can consider at different stages of a patient’s oncological journey, either as a standalone treatment or in combination with systemic therapy [12,13,14,15,16]. The oligometastatic setting is defined as an intermediate disease status, falling between localized and polymetastatic, where local treatment to all active metastatic sites could potentially lead to a cure.

Over the last decade, technological advancements have greatly increased clinicians’ confidence in proposing local ablative treatments. This progress has pushed the boundaries not only in terms of target numerical cut-offs but also by introducing novel clinical indications for sites traditionally deemed unsuitable for radiotherapy [17,18,19,20]. Historically, the role of radiotherapy for abdominal disease was largely confined to palliative treatments delivered to the entire abdomen, which yielded poor results and high toxicity rates, even with intensity-modulated image-guided radiotherapy (IMRT-IGRT) [21].

### 1.4. Challenges and Future Directions in Peritoneal Radiotherapy

Treating both peritoneal disease and abdominal wall recurrences presents a unique challenge for radiation oncologists due to the close proximity of intestinal loops. Delivering safe and effective treatment to such a critical anatomical site requires a constant balance between safety and efficacy for the oncological patient.

Isolated peritoneal nodules are remarkably uncommon. To date, only a single case report by Boldrini et al. has documented outcomes of a peritoneal metastasis treated with SBRT [22]. In light of this, our current series aims to present the outcomes from a single-institution cohort of patients who received SBRT for isolated peritoneal metastases and abdominal wall recurrences.

## 2. Methods

This study presents a single-institution retrospective analysis of stereotactic body radiotherapy (SBRT) for oligometastases located in the peritoneum or abdominal wall.

All participating patients provided written informed consent. Due to the retrospective, single-institution nature of the study and the absence of any experimental treatments, ethical committee approval was not required. Furthermore, full adherence to the European General Data Protection Regulation (GDPR) was maintained for all patients.

The current study compiles outcomes for a total of 26 lesions across 20 patients who received SBRT for oligometastatic disease within the peritoneum or abdomino-pelvic wall. We categorized these cases into three types of oligometastatic disease—oligorecurrent, oligoprogressive, or oligopersistent—following the definitions provided by the ESTRO-EORTC consensus [12].

In this series, all treated patients had undergone at least one cytoreductive surgery and one course of intraperitoneal chemotherapy. The potential utility of radiotherapy was thoroughly discussed by a multidisciplinary team and evaluated on a case-by-case basis. Radiotherapy was considered a viable treatment option for patients with a numerically limited disease presentation who were not candidates for other local therapies.

### 2.1. Patient Selection and Treatment Planning

Inclusion criteria for this study were as follows:

- ECOG Performance Status (PS) ≤ 2;
- Signed written informed consent;
- A maximum of five lesions treated simultaneously;
- Radiotherapy schedules applying a minimum dose of 6 Gy per fraction.

A 2 mm thickness CT scan was performed with the patient in the supine treatment position, immobilized using an abdomino-pelvic thermoplastic mask and a knee–ankle immobilization device. Additionally, patients were requested to fast for three hours, depending on the lesion’s location and its proximity to critical healthy structures like the stomach.

To enhance target volume delineation accuracy, PET-CT imaging (or MRI when available) was merged with the treatment planning CT scan for all patients. The Gross Tumor Volume (GTV) was considered equal to the Clinical Target Volume (CTV). The Planning Target Volume (PTV) was generated by adding a 3 mm margin in all directions, except for the cranio-caudal direction, where a 3–5 mm margin was applied at the physician’s discretion. Treatment planning aimed to ensure at least 95% coverage of the PTV by 95% of the prescribed dose, without compromising dose constraints for organs-at-risk.

### 2.2. Radiotherapy Delivery and Follow-Up

All patients received image-guided SBRT using Helical Tomotherapy (Accuray, Sunnyvale, CA, USA). Daily image guidance involved a megavoltage CT scan performed before each treatment session.

Following the final radiotherapy session, follow-up appointments were scheduled every 3–4 months for the first two years. For patients without progressive disease, follow-up transitioned to every 6 months starting from the third year.

### 2.3. Endpoints and Statistical Analysis

The primary endpoint of the study was local control (LC), defined as the time from the end of treatment to either an in-field relapse or the last follow-up time.

Secondary endpoints included the following:

- Distant progression-free survival (DPFS): The interval between the end of treatment and the occurrence of disease relapse outside the radiotherapy field or the last follow-up time.
- Time to next systemic treatment (TNST): The interval between the end of treatment and the initiation of a new line of systemic treatment or the last follow-up time.
- Polymetastatic-free survival (PMFS): Measured from the end of treatment to the onset of polymetastatic dissemination or the last follow-up time.
- Overall survival (OS): The interval between the end of treatment and death from any cause or the last follow-up time.

Toxicity assessment for acute and late adverse events was performed according to the Common Terminology for Adverse Events (CTCAE) criteria v5.0. Acute toxicity was defined as any adverse event occurring within 90 days from the end of treatment or during radiotherapy, while late toxicity encompassed any adverse event occurring after 90 days from the end of treatment.

Categorical variables were summarized as counts and proportions, while continuous variables were described using measures of central tendency (means and medians) and dispersion (standard deviations and ranges), as appropriate.

To explore associations between categorical variables, Fisher’s exact test was applied based on expected cell counts. The Kruskal–Wallis test was used to assess differences among continuous or ordinal variables across independent groups.

Kaplan–Meier estimates were used to calculate time-to-event outcomes, including local control (LC), distant progression-free survival (DPFS), polymetastatic-free survival (PMFS), time to next systemic treatment (TNST), and overall survival (OS). Survival curves were generated for categorical variables, and log-rank tests were employed to compare distributions across subgroups. Median survival times were estimated using the product-limit (Kaplan–Meier) method, while median follow-up time was calculated using the reverse Kaplan–Meier method.

All statistical analyses were performed using R Statistical Software (version 4.2.3; R Core Team, 2023). A *p*-value ≤ 0.05 was considered statistically significant. As this was an exploratory analysis in a small cohort, *p*-values were not adjusted for multiple testing and results should be interpreted accordingly.

## 3. Results

Between April 2020 and September 2024, a total of 26 oligometastatic lesions located in the peritoneum or in the abdominal wall detected in 20 patients received SBRT with Helical Tomotherapy. Only in three patients, out of twenty, did more than one lesion receive SBRT: two lesions in two patients, and five lesions in a single case of colorectal cancer with ongoing third line systemic treatment. The median total dose was 30 Gy (27–35 Gy) in five fractions (3–5). The most frequent primary neoplasm was ovarian cancer in 14/20 cases, endometrial in 2/20 cases, while the remaining were colorectal, vaginal, pancreatic and non-small cell lung cancer. Four lesions were located in the abdominal wall, while the remaining twenty-two were located in the peritoneum. Concurrent systemic therapy was administered in 18/20 patients, consisting of PARP-inhibitors in 14 cases, chemotherapy in 3 cases, and immunotherapy in 1 case of metastatic non-small cell lung cancer (Table 1).

**Figure 1:**
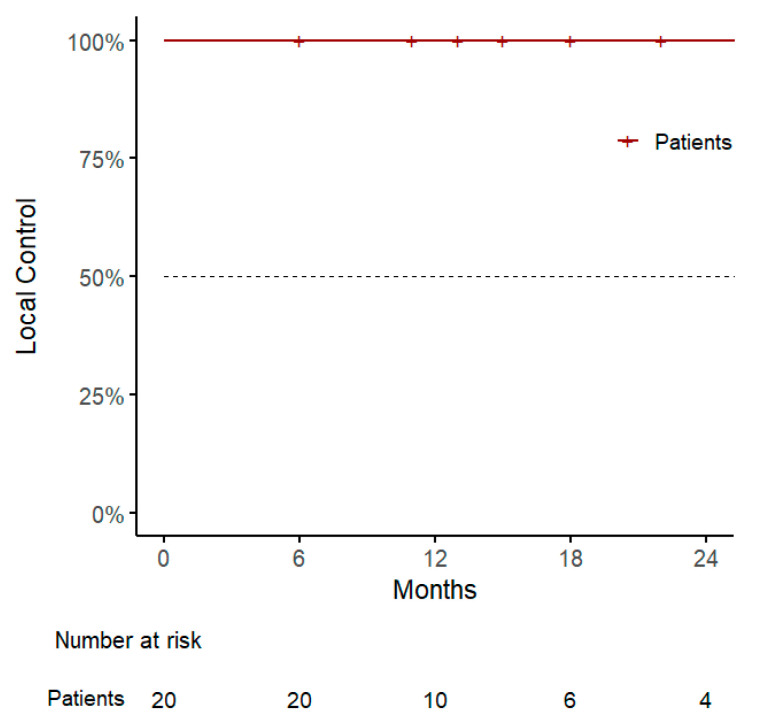
Kaplan–Meier curve for local control.

With a median follow-up of 15 months (range, 6–59), our 1-year LC was 100% (95% CI: 100–100%), with two local failures reported after 28 and 31 months, respectively, and both treated with a second course of SBRT (32.5 Gy in 5 sessions) (Figure 1).

Also, in the case of re-irradiation, treatment was well tolerated and the patients continued their ongoing systemic therapy up to the last known follow-up.

A statistically significant association was observed between the number of prior surgical procedures and improved local control (*p* = 0.04), as assessed using the Kruskal–Wallis test (Table 2).

**Figure 2:**
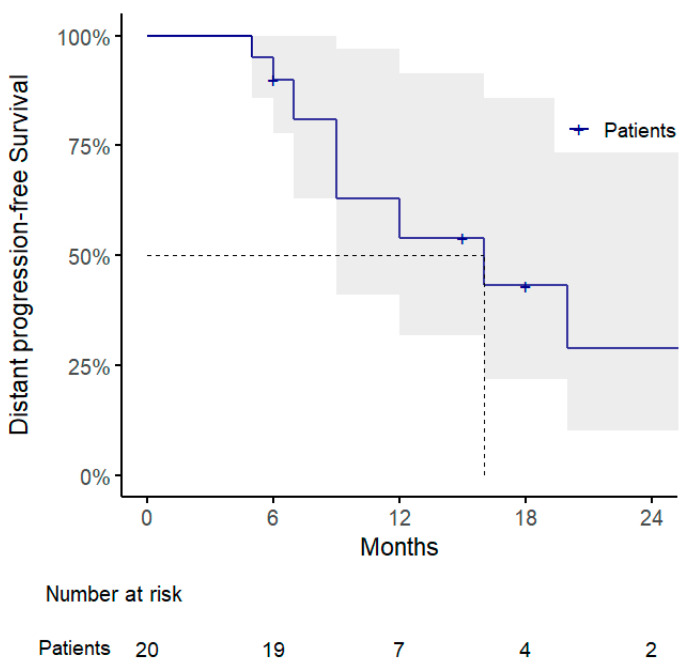
Kaplan–Meier curve for distant progression-free survival.

**Figure 3:**
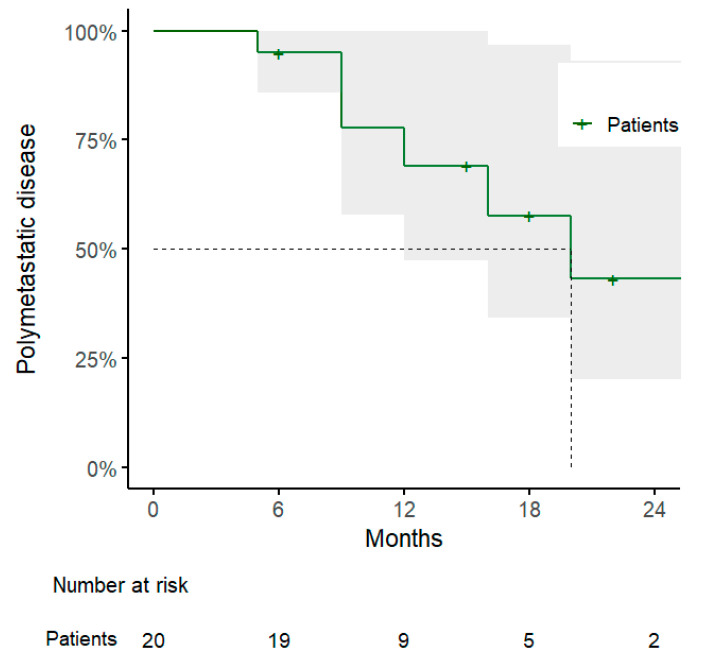
Kaplan–Meier curve for polymetastatic disease-free survival.

Regarding the other clinical outcomes, 1-year DPFS and PMFS rates were 54% (95% CI: 32–91%) and 69% (95% CI: 48–100%), with a pattern of progression consisting of polymetastatic spread in 7/10 cases, of which 3 showed evidence of disseminated peritoneal carcinosis and were considered candidates for best supportive care (Figure 2 and Figure 3 and Table 3).

Three patients received a further course of SBRT due to repeated oligoprogression, after 22, 28 and 31 months, respectively.

**Figure 4:**
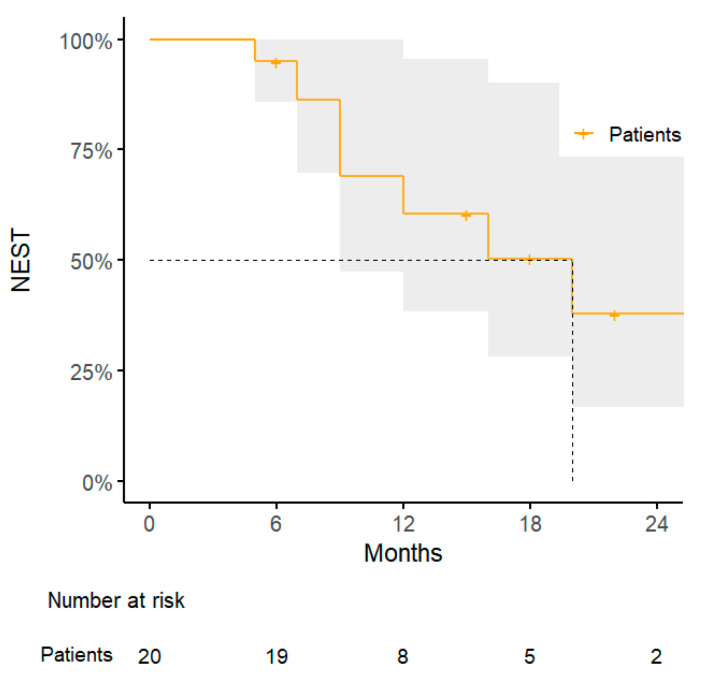
Kaplan–Meier curve for next systemic treatment-free survival.

The administration of SBRT for peritoneal/abdominal wall oligometastases lead to a 1-year TNTS rate of 61% (95% CI: 38–96%), with a median time to next systemic treatment of 8 months (range, 6–47 months) (Figure 4).

**Figure 5:**
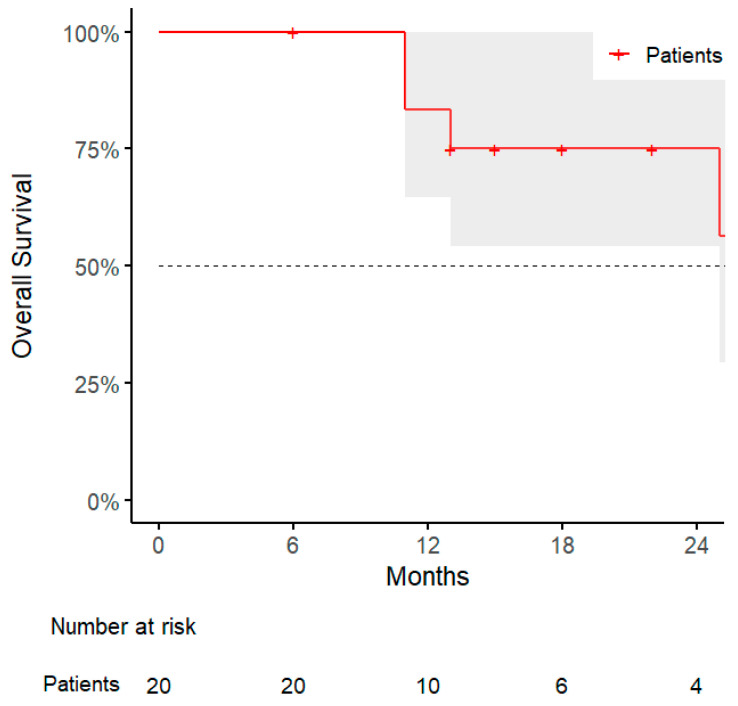
Kaplan–Meier curve for overall survival.

The median overall survival (OS) was 12 months (range, 6–59), with a 1-year OS rate of 83% (95% CI: 65–100%, Figure 5). Statistical analysis revealed that patients with abdominal wall lesions had significantly improved OS compared to those with peritoneal lesions, with a significant association identified using Fisher’s exact test (*p* = 0.03), and confirmed by log-rank test (*p* = 0.01). Similarly, patients who experienced sequential oligometastatic progression and were treated with a second course of SBRT showed improved OS; the association was significant (*p* = 0.04), and survival benefit was confirmed by log-rank analysis (*p* = 0.04) (Table 4, Table 5 and Table 6).

Finally, a prolonged efficacy of the ongoing systemic therapy was also significantly associated with better OS (*p* < 0.01), further supporting the role of local treatments in sustaining systemic control.

No G ≥ 3 adverse events occurred. Two patients reported acute G1 abdominal pain after SBRT, and one case of late G2 abdominal pain successfully treated with one-week steroid therapy was observed in a patient who received a stereotactic re-irradiation of the same peritoneal nodule after 28 months from the first course of treatment. Excellent tolerance to the treatment was observed for the single case who received SBRT simultaneously to five peritoneal nodules with no relevant adverse events.

## 4. Discussion

To the best of our knowledge, this is the first study reporting the outcomes of a series of patients treated with Helical Tomotherapy-based SBRT for lesions located in the peritoneum or in the abdomino-pelvic wall. Despite the relatively small sample size, to date, the use of stereotactic radiotherapy for peritoneal metastases has been reported only in case reports.

Smile et al. reported the outcomes of 34 patients who received SBRT to abdomino-pelvic oligometastases arising from gynecological malignancies, in a mixed series of lymph-nodal and soft tissue metastases (no peritoneal metastases) reporting promising rates of LC at 1 and 2 years, both of 92.5%, with excellent tolerability [23].

The authors focused on the role of SBRT as a helpful tool for patients who develop a disease recurrence close to the surgical access, where limited options are available, especially for patients who have been heavily treated with repeated surgical procedures.

In this scenario, SBRT offers a non-invasive alternative able to deliver focused high doses to small volumes, without compromising the abdominal wall or the adjacent bowel loops.

As limited data are available for abdominal wall recurrences, the use of stereotactic radiotherapy for peritoneal metastases is even more anecdotal.

Boldrini et al., at first, published a case report of a single patient treated with MR-guided SBRT for a solitary peritoneal nodule from colorectal cancer [22].

This case report highlighted the potential role of daily adapted radiotherapy, due to the ability to edit target volume delineation and treatment plan based on real-time anatomy, with a consequent improved accuracy in treatment delivery and healthy structure sparing, as also reported in other MR-guided SBRT experiences for abdomino-pelvic lesions [24].

More recently, Romano et al. reported the preliminary outcomes of a retrospective cohort of 34 patients treated with MR-linac for peritoneal oligometastases, with promising data in terms of LC and no incidence of severe adverse events [25].

As daily adaptive radiotherapy might represent the theoretically preferred treatment option in the case of targets exposed to the issues of organ motion, abdominal targets are often a site of concern for radiation oncologists due to the close proximity of intestinal loops and the need to also consider the potential motion of both lesions and OARs [26].

Moreover, another concern is represented by patient tolerance, which might be affected by the personal history of heavy pre-treatments, not only in terms of systemic therapy (chemotherapy, anti-angiogenic agents, etc.) but also in terms of surgical interventions.

Nonetheless, our series reports the safety of a treatment delivered with conventional linacs for peritoneal oligometastases, as a result of a careful multidisciplinary team evaluation analyzing the natural history of the disease, the anatomical location of the target lesion and its potential shifts due to organ motion.

Also, the feasibility of SBRT treatment was evaluated by considering the possibility of using nearby healthy organs, such as the psoas muscle or bony structures, which are less susceptible to organ motion, as reference points. This strategy simplified the process of daily image-guidance for positioning verification, leading to a safer treatment delivery.

The attractiveness of SBRT in this scenario relies on the possibility to extend the ongoing systemic treatment delaying the switch to the subsequent therapeutic line, especially in the case of gynecological malignancies, where limited options are available, as reported in a multicentre retrospective experience of patients with ovarian cancer treated with SBRT for oligoprogressive disease with ongoing PARP-inhibitors [27].

These data find confirmation in our series, where patients treated with SBRT for repeated oligoprogressions maintain a survival advantage compared to those who develop a polymetastatic dissemination, and, consequently, prolonged efficacy of a line of systemic therapy relates to improved OS.

Notably, our series reports excellent preliminary data in terms of LC, with two late local failures that occurred after 28 and 31 months, respectively. Both of these failures, after a careful assessment of the initial treatment plan, have been managed with stereotactic re-irradiation, due to the relatively long interval between the two courses of SBRT; thus, stereotactic radiotherapy shows an attractive versatility not only for the treatment of oligometastases located in critical sites, but also in the case of previous radiotherapy treatments, as initial promising experiences are reporting [28].

However, peritoneal metastases still have a detrimental impact on OS, as our series reports a survival advantage for patients who relapsed in the abdominal wall, compared to the peritoneum. Nonetheless, the role of local treatments needs to be further explored in this peculiar anatomical setting, traditionally considered not suitable for metastasis-directed therapies, and particularly for radiotherapy.

This study has several limitations: first of all, the small sample size and the retrospective nature of the series affect the statistical power of the analysis; also, there is a substantial heterogeneity in terms of lesion site, primary histology and concurrent systemic treatments. Furthermore, due to the limited literature data, we cannot determine the optimal dose for peritoneal oligometastases, despite the promising LC rates reported in this series.

Despite being a relatively small sample, it currently represents the largest series in the literature of patients treated with stereotactic body radiotherapy for oligometastases located in the peritoneum or in the abdominal wall.

Future studies with larger cohorts and prospective design might help clinicians to develop this treatment option for patients suitable for stereotactic radiotherapy.

Nonetheless, this is the first series reporting the outcomes of patients treated with SBRT to peritoneal nodules, highlighting the promising tolerability of this treatment option and the results in terms of local control, suggesting a potential new therapeutic approach in the case of oligometastatic presentation.

## 5. Conclusions

This study reports excellent promising outcomes when SBRT is applied for the treatment of solitary peritoneal nodules in carefully selected patients, thus supporting the role of local ablative therapies as a potential therapeutic tool able to postpone the switch to a new systemic agent. Larger cohort studies and prospective experiences are warranted to assess optimal patients’ selection criteria and the real impact of SBRT in the natural history of the disease for this subset of patients.

## Figures and Tables

**Table 1:** Patients characteristics.

Patients Characteristics	N or Median Value (Range)
Age; M:F	Age = 62 years (range, 41–78 years); M:F = 1:19
Primary Site	ovarian cancer = 14/20; endometrial cancer = 2/20; non-small cell lung cancer = 1/20; pancreatic cancer = 1/20; vaginal cancer = 1/20; colorectal cancer = 1/20
Primary Histology	serous cell = 14/20; papillary = 1/20; adenocarcinoma = 3/20; squamous cell = 1/20; adenosquamous = 1/20
N. of surgical interventions	2 (range, 0–5)
Disease-free interval	24 months (range, 0–158)
Abdominal site of oligometastases	Abdominal wall = 4; peritoneum = 22
N. of treated lesions with SBRT	1 (range, 1–5)
Concurrent systemic therapy	18/20
GTV and PTV	GTV = 3.85 cc (range, 1.8–39.8 cc); PTV = 7.25 cc (range, 3.1–51.7 cc)
SBRT total dose	30 Gy (range, 27–35 Gy) in 5 fx (range, 3–5)

Abbreviations: GTV = gross tumor volume; PTV = planning target volume; SBRT = stereotactic body radiotherapy.

**Table 2:** Univariate Analysis of Factors Associated with Local Control by Fisher’s exact test. Local Control (LC) was defined as the absence of in-field recurrence after stereotactic body radiotherapy. Sequential oligometastatic progression is defined as the need for a second course of SBRT for newly appearing oligometastases.

Parameter	Outcome		*p*-Value
	Local control		
	Yes (18)	No (2)	
Abdominal location			1
Abdominal wall	4 (22.22%)	0 (0%)	
Peritoneum	14 (77.78%)	2 (100%)	
Nest			1
No	11 (61.11%)	1 (50%)	
Yes	7 (38.89%)	1 (50%)	
Polymetastatic disease			1
No	12 (66.66%)	2 (100%)	
Yes	6 (33.33%)	0 (0%)	
Sequential oligometastatic			0.07
No	7 (38.89%)	0 (0%)	
Yes	1 (5.55%)	2 (100%)	

Abbreviations: NEST: initiation of new systemic therapy.

**Table 3:** Univariate analysis of factors associated with status by Fisher’s exact test. Polymetastatic progression was defined as the transition from a limited (oligometastatic) disease burden to widespread metastatic dissemination (e.g., >5 lesions or diffuse peritoneal carcinomatosis). Sequential oligometastatic progression is defined as the need for a second course of SBRT for newly appearing oligometastases.

Parameter	Outcome		*p*-Value
	Polymetastatic		
	No (14)	Yes (6)	
Abdominal location			0.06
Abdominal wall	1 (7.14%)	3 (50%)	
Peritoneum	13 (92.86%)	3 (50%)	
Sequential oligometastatic			**0.03**
No	1 (7.14%)	6 (100%)	
Yes	3 (21.42%)	0 (0%)	

**Table 4:** Univariate analysis of factors associated with status by Fisher’s exact test or Kruskal–Wallis. Status refers to the overall survival condition at last follow-up (Alive vs. Dead). Sequential oligometastatic progression is defined as the need for a second course of SBRT for newly appearing oligometastases.

Parameter	Outcome		*p*-Value
	Status		
	Alive (15)	Dead (5)	
Abdominal location			**0.03**
Abdominal wall	1 (6.66%)	3 (60%)	
Peritoneum	14 (93.3%)	1 (20%)	
Nest			**<0.001**
No	12 (80%)	0 (0%)	
Yes	3 (20%)	5 (100%)	
Polymetastatic			**<0.001**
No	14 (93.3%)	0 (0%)	
Yes	1 (6.66%)	5 (100%)	
DPFS			
No Progression	11 (73.33%)	0 (0%)	**0.01**
Progression	4 (26.67%)	5 (100%)	
Number of lesions	1(1–2)	1 (1–1)	0.06

Abbreviations: NEST: initiation of new systemic therapy; DPFS = distant progression-free survival.

**Table 5:** Univariate analysis of factors associated with status by Fisher’s exact test. Distant Progression-Free Survival (DPFS) was defined as the interval between the end of SBRT and the appearance of new metastatic lesions outside the treated field. Sequential oligometastatic progression is defined as the need for a second course of SBRT for newly appearing oligometastases.

Parameter	Outcome		*p*-Value
	DFPS		
	No Progression (11)	Progression (9)	
Abdominal location			0.28
Abdominal wall	0 (0%)	3 (33.33%)	
Peritoneum	10 (91%)	6 (66.66%)	
Nest			**<0.01**
No	11 (100%)	1 (11.11%)	
Yes	0 (0%)	8 (88.89%)	
Polymetastatic			**<0.01**
No	11 (55%)	3 (15%)	
Yes	0 (0%)	6 (30%)	
Sequential oligometastatic			0.3
No	0 (0%)	7 (77.77%)	
Yes	1 (9.09%)	2 (22.22%)	

Abbreviations: NEST: initiation of new systemic therapy.

**Table 6:** Univariate analysis of factors associated with status by Fisher’s exact test. NEST was defined as the initiation of a new line of systemic therapy following SBRT. Sequential oligometastatic progression is defined as the need for a second course of SBRT for newly appearing oligometastases.

Parameter	Outcome		*p*-Value
	Nest		
	No (12)	Yes (8)	
Abdominal location			0.61
Abdominal wall	1(8.33%)	3 (37.5%)	
Peritoneum	11 (91.67%)	5 (62.5%)	
Polymetastatic			**<0.001**
No	12 (100%)	2 (25%)	
Yes	0 (0%)	6 (75%)	
Sequential oligometastatic			0.07
No	0 (0%)	7 (87.5%)	
Yes	2 (16.66%)	1 (12.5%)	

Abbreviations: NEST: initiation of new systemic therapy.

## Data Availability

Data are contained within the article.
